# Rat Glioma 101.8 Tissue Strain: Molecular and Morphological Features

**DOI:** 10.3390/ijms26188992

**Published:** 2025-09-15

**Authors:** Anna Igorevna Alekseeva, Alexandra Vladislavovna Sentyabreva, Vera Vladimirovna Kudelkina, Ekaterina Alexandrovna Miroshnichenko, Alexandr Vladimirovich Ikonnikov, Elena Evgenievna Kopantseva, Anna Mikhailovna Kosyreva, Timur Khaysamudinovich Fatkhudinov

**Affiliations:** 1 Avtsyn Research Institute of Human Morphology, Federal State Budgetary Scientific Institution ‘Petrovsky National Research Center of Surgery’, 117418 Moscow, Russia; 2 Research Institute of Molecular and Cellular Medicine, Peoples’ Friendship University of Russia (RUDN University), 115093 Moscow, Russia

**Keywords:** glioblastoma, experimental rat glioma, brain tumor, scRNA-seq, tumor microenvironment

## Abstract

The search for markers applicable for efficient differential diagnosis and personalized therapy is a priority task of experimental neuro-oncology. Modern molecular methods allow us to analyze human biopsy material; however, further actions with this extracted tumor tissue are limited. Relevant and sophisticated CNS tumor models are required for precise therapy development. Although it is possible to use human biomaterial to create 2D and 3D cultures and implant them into xenograft animals, the data generated from such models is limited. Due to changes in the classification of the CNS tumors in 2021, a representative model should have not only morphological similarity to human tumors but also key genetic aberrations for studying the mechanisms of carcinogenesis and personalized therapy (such as PDGFRa, Olig1/2, Sox2, and Mki67) for different glioma models such as astrocytoma, oligodendroglioma, and glioblastoma. On the basis of a unique scientific facility “The Collection of experimental tumors of the nervous system and neural tumor cell lines” (Avtsyn Research Institute of Human Morphology of “Petrovsky National Research Center of Surgery”), there is a biobank of chemically induced transplantable tumors of laboratory animals. Their properties, mechanisms, and progression closely correlate with malignant CNS neoplasms in humans. These are potentially useful for identifying novel signaling pathways associated with oncogenesis in the nervous system and personalizing therapeutic approaches. In our work, we characterized a tissue-transplantable brain tumor strain of rat glioma101.8 using MRI, IHC, scRNA-seq, and qPCR-RT methods. According to this study, the cellular composition of the tissue-transplantable rat glioma 101.8 strain was determined, as well as the major genetic signature characteristics of each cell population of this tissue-transplantable strain and its microenvironment.

## 1. Introduction

Diffuse gliomas are malignant brain neoplasms of astrocyte and oligodendroglia origin [1]. Depending on the tumor type, the prognosis of life expectancy after standard therapy varies from relatively favorable ones with decades-long survival to extremely poor ones within several months. For low-grade gliomas (grade 2) such as astrocytoma and oligodendroglioma, median patient survival after standard therapy is about 65 months and 139 months, respectively, with 5-year survival rates of 74–90% and over 80% [2]. Patients with glioma grade 3–4 have median survival durations of 45 months for astrocytoma and 65 months for oligodendroglioma after standard therapy. Glioblastoma and astrocytoma are the most aggressive subtypes of malignant diffuse gliomas in adults (grade 4). The median survival of patients is 13–15 and no longer than 24 months for glioblastoma [3], 52 months for astrocytoma, and 76 months for oligodendroglioma after standard therapy. Neural stem cells (Nestin^+^, SOX2^+^) are considered as possible cell progenitors of glioblastoma, oligodendroglioma, and astrocytoma, while oligodendrocyte progenitor cells (Olig2^+^, PDGFRα^+^) give rise glioblastoma and oligodendroglioma, and astrocytes (GFAP^+^) are possible progenitors of glioblastoma and astrocytoma.

Therapy for diffuse glioma consists of a combination of surgical, chemotherapy, and radiotherapy approaches. For highly malignant glioma, only resection of more than 99% (gross-total resection) of the tumor leads to a significant increase in the life expectancy of such patients. Otherwise, the use of therapeutic methods alone shows low efficacy [4]. The existing therapy approaches are mainly represented by classical cytostatic, such as the “gold standard therapy” temozolomide in combination with second-line drugs like procarbazine, lomustine, vincristine, paclitaxel, etc. [5]. Novel drugs are quite a challenge to bring in clinical practice. For instance, the antiangiogenic drug bezacizumab, although showing good results, does not lead to a significant increase in life expectancy [6]. So, the search for new effective methods of therapy and the development of new drugs remain a priority task for modern science.

One of the directions of experimental neuro-oncology is precision therapy aimed at specific molecular targets. Therefore, it seems relevant to develop experimental tumor models with a specific expression profile of molecular markers suitable for the further evaluation of precision therapy drugs [7].

In 2021, an updated WHO CNS5 classification was published. According to it, differential diagnosis is now predominantly based on molecular markers rather than morphologic features [8]. Thus, the key markers for the differentiation of diffuse glioma into oligodendroglioma, astrocytoma, and glioblastoma were the presence of IDH mutation and the presence of 1p/19q codeletion [9]. The presence of other molecular mutations is only used for additional tumors despite their high occurrence frequency. However, they can be used as targets for precision therapy. Such mutations include EGFR amplification (found in about 50% of grade 4 primary glioblastoma cases) [10]; ATRX, PTEN, TP53, CDKN2A/B, and TERT promoter mutations; and MGMT promoter methylation, as well as aberrations of chromosomes 7 and 10 [11].

The tumor microenvironment (TME) is a promising target for tumors therapy, since it actively supports tumor homeostasis, influences progression, and mediates the response to treatment. It includes macrophages/microglia, endotheliocytes, fibroblasts, lymphocytes (including NK cells), and neural progenitor cells [12]. The colonization of the tumor and peritumoral zone by cytotoxic T lymphocytes (CTLs) is a frontier search direction of immunotherapy, CAR-T in particular. In different types of gliomas (glioblastoma, astrocytoma, and oligodendroglioma), the TME has specific features such as high infiltration by tumor-associated macrophages/microglia (TAMs), pronounced neoangiogenesis with abnormal vessels, hypoxia, and powerful immunosuppression, especially in aggressive forms such as glioblastoma [13].

The development of effective precision therapy approaches for the CNS tumors requires relevant and reproducible experimental models that correspond highly to the complex spontaneous neoplasm biology. Although human biopsy material is actively used to create 2D/3D cultures and xenographic models on immune-deficient animals, these approaches have significant methodological limitations [14]. In vitro cell cultures do not possess pharmacokinetics, so they cannot elucidate the effects of the tumor microenvironment (TME) and systemic interaction features in vivo, despite their usefulness for cytotoxicity screening and other research purposes. These factors do not allow us to evaluate any therapeutic efficacy in vivo. Xenographic models suffer from a lack of a functional host immune response and inadequate human-specific TME reconstruction, including key immune–stromal interactions of the blood–brain barrier [15]. Therefore, syngeneic (allogeneic) tumor models in immune-competent animals appear to be most relevant for experimental neuro-oncology, since they provide an intact immune system and a physiological microenvironment, which are critically important for translational research.

Experimental rat glioma 101.8 is a transplantable tumor tissue strain. The primary tumor was induced by the carcinogen 9,10-Dimethyl-1,2-benzanthracene (DMBA) in sexually mature female rats with subsequent transplantations of tumor tissue fragments. As a result, the tumor acquired a stable histological structure and retained a heterogeneous structure due to the stromal component [16]. Glioma 101.8 is used to investigate the biology of carcinogenesis and test diagnostic and therapeutic methods [17,18]. According to its histologic characteristics, glioma 101.8 corresponds highly to human glioblastoma. The tumor is characterized by high cellularity, multiple pseudopalisade necroses, and perineuronal and perivascular growth. Previously, we provided a comparative morphological and molecular characterization of glioma 101.8 compared to two other tissue strains of experimental rat glioma depending on the carcinogen used [19]. At the same time, the tumor and its microenvironment molecular landscape remain unexplored. Identification of tumor cell subpopulations, as well as analysis of its microenvironment clusters alongside the main signaling pathways and key genetic signatures for this tissue strain, will not only prove the relevance of translational results but also allow the develop of target therapy approaches. The purpose of this work was to integrally assess the main features of rat glioma tissue strain 101.8 and its microenvironment via MRI imaging, histological and ICH analysis, qPCR-RT, and single-cell sequencing.

## 2. Results

### 2.1. MRI Study of the Rats’ Brain with Transplanted Glioma 101.8

**Figure 1:**
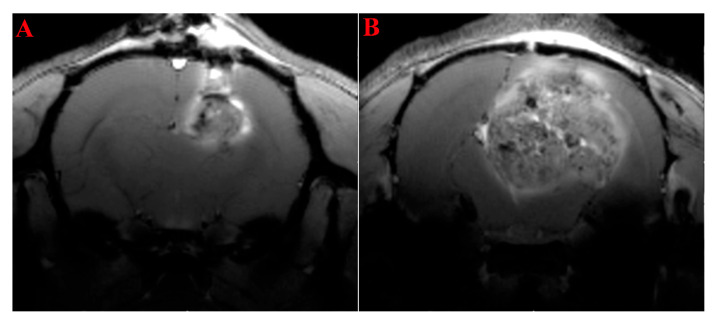
T_1_-weighted MRI images of the brains of rats with glioblastoma 101.8 on days 7 (**A**) and 18 (**B**) after transplantation using the Gd-containing contrast agent Gadovist^®^.

According to the MRI study on day 7 and day 18 after implantation of the rat glioma 101.8 tissue strain, there was an uneven accumulation and distribution of the contrast agent, with areas of high and reduced contrast, indicating a heterogeneous tumor structure (Figure 1). On day 7 after tumor implantation, higher contrast agent accumulation was noted in the peritumoral zone, which probably occurred due to the presence of peritumoral post-operative inflammation (Figure 1A). Meanwhile, on day 18 of rat glioma 101.8 growth, contrast accumulation at the tumor border became lower. The tumor itself was localized strictly within the brain tissue, but it had a slight bulge along the track through the skull opening. The development of foci separate from the main tumor was not observed.

For the tissue model of glioma, the pattern of contrast agent accumulation is irregular. There were zones of reduced contrast accumulation, which are typical of necrosis zones (Figure 1B). The borders of the tumor were clear, with a slight edema in the peritumoral area.

### 2.2. Histologic Characterization of GB 101.8

**Figure 2:**
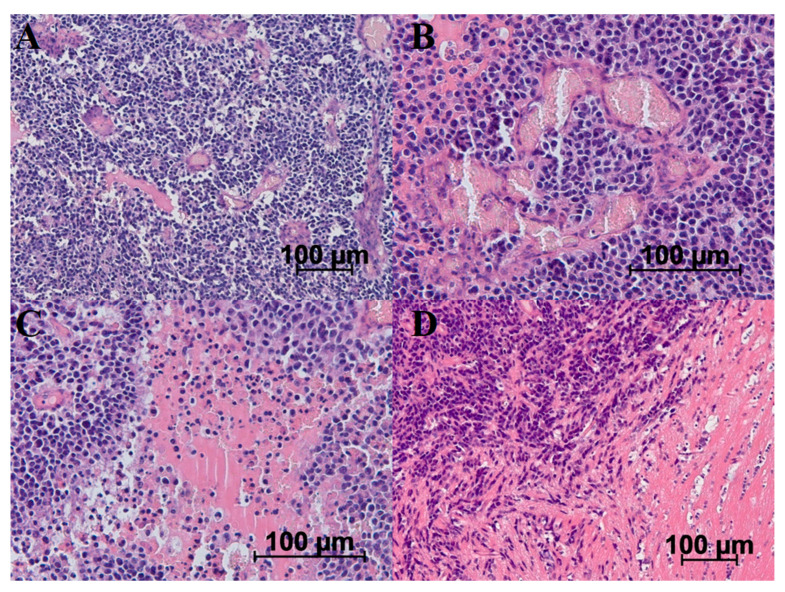
Morphological features of rat glioma 101.8 on the 18th day after implantation. (**A**) Polymorphic tumor cells and multiple de novo vessels with thin unformed walls with necroses areas. (**B**) Group of de novo vessels. (**C**). (**D**) Necroses. The boundary of the tumor and healthy tissue is focally indistinct, indicating invasion of tumor cells into healthy tissues. Hematoxylin and eosin staining.

Regarding histological preparations of rat glioma 101.8, tumor cells had a polymorphic shape with polymorphic nuclei and a wide rim of cytoplasm. Mitotically dividing cells were identified in 1–2 fields of view at ×400. Both on the periphery and in the central zone of the tumor, a large number of newly formed vessels with thinned and partially formed walls were detected. A large number of extensive necroses and hemorrhages were observed throughout the tumor area. Peritumoral edema and glial shafts of astrocytes were detected along the tumor margin. The border between the tumor and healthy tissue was discontinuous, with areas of perineuronal and perivascular tumor invasion into healthy tissue (Figure 2).

### 2.3. Characterization of Rat Glioma 101.8 Cell Subpopulations and Their Microenvironment

**Figure 3:**
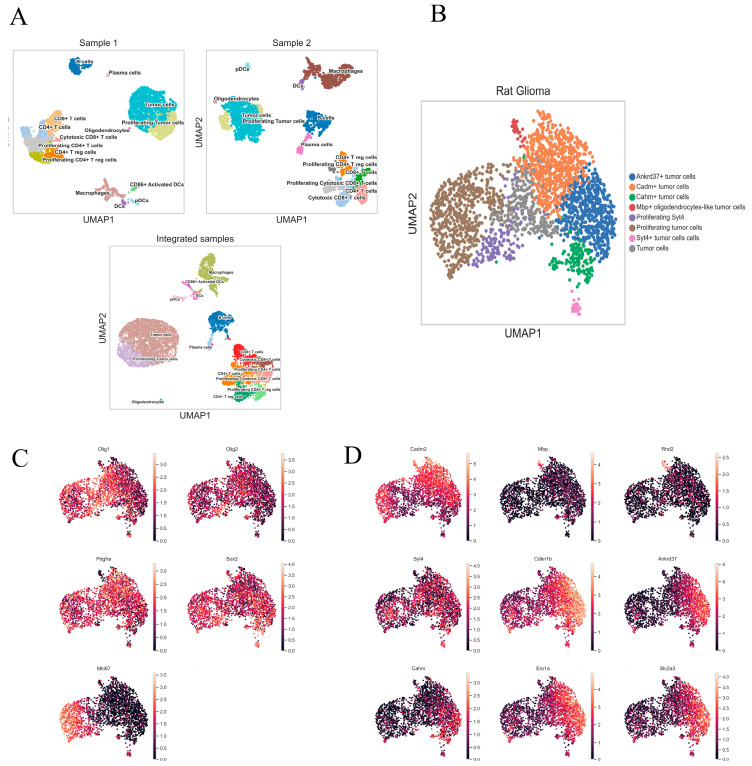
(**A**) UMAP plot of GB 101.8 samples. Sample 1, Sample 2 and integrated plot of two rat glioma 101.8 tissue strain samples; (**B**) Representations of the *Olig1*, *Olig2*, *Pdgfra*, *Sox2*, and *Mki67* genes in the rat glioma 101.8 tumor cells cluster (integrated analysis); (**C**) Annotated subclustering of tumor subpopulations in the rat glioma 101.8 tissue strain; (**D**) Top 9 most expressed genes characterizing tumor subclusters of the rat glioma 101.8 tissue strain.

According to transcriptome analysis of scRNA-seq gene expression data, cell populations constituting the tumor microenvironment included aredendritic cells, macrophages, B cells, T cells, and oligodendrocytes (Figure 3). The tumor cell population was distinguished based on the results of aneuploidy analysis on the obtained CAN matrix.

Based on transcriptional profile data, the T-cell cluster was divided into subpopulations of CD4^+^ T-helper cells, CD8^+^ T-cytotoxic cells, regulatory T cells, and actively proliferating T cells, which were characterized by increased expression of *Mki67*. Among dendritic cells (DCs), plasmacytoid dendritic cells (pDCs) expressed *Bst2*, *Tsf4*, *Irf7*, and *Ly6c* at high levels. Dendritic cells expressing *ClEc9a*, *Cadm1*, *Xcr1*, *Itgax*, *Ccr7*, and *Cd80* and a minor population of Cd86^+^ dendritic cells were detected.

Sixteen (16) cell clusters were detected in rat glioma 101.8 samples. The population composition of each cluster and the major expressed signaling pathways in each cluster were identified based on manual typing for the most highly expressed genes, identification of signaling pathways, and biological processes, as well as karyotyping (Table 1, Tables S1 and S2, and Figure 3C).

### 2.4. Integrated Heterogeneity Analysis of the Tumor Cluster

A tumor cells cluster was identified by including cells with high expression of the following genes: *Mki67*, *Pcna* (proliferating cell nuclear antigen), and *Top2a* (DNA topoisomerase II alpha) (proliferation markers); *Sox2* (SRY-box transcription factor 2) and *Nes* (Nestin) (stem cell markers); oligodendrocyte transcription factor 2 *Olig2*; and *Pdgfra* (platelet-derived growth factor receptor A) as a vascularization marker. In this work we analyzed the representation of cells with high expression of diffuse glioma signature genes, more specifically *Olig1*, *Olig2*, *Pgfra*, *Sox2*, and *Mki67* (Figure 3B).

Tumor cell subclusters were identified to assess the intrapopulation heterogeneity of the tumor. Highly expressed genes were identified by comparing their expression with averaged values in other tumor subclusters. Further, we obtained data on the top 10 genes expressed in the tumor cell subclusters (Table S3).

The analysis identified nine (9) cell clusters, two of which were combined into one, namely “tumor proliferating cells” (Figure 3C).

The nine (9) most expressed genes characterizing tumor heterogeneity were identified as follows: cell adhesion molecule 2 (*Cadm2*), myelin basic protein (*Mbp*), Rho-GTPase family member (*Rnd2*), synaptotagmin (*Syt4*), cyclin-dependent kinase 1B inhibitor (*Cdkn1b*), ankyrin repeat domain 37 (*Ankd37*), long non-coding RNA (*Cahm*), endoplasmic reticular oxygen reductase 1 alpha (*Ero1a*), and transmembrane glucose transporter protein (*Slc2a3*) (Figure 3D).

**Figure 4:**
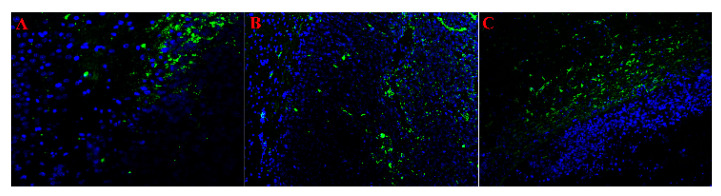
ICH staining using antibodies to Sox2 ((**A**), ×400), Olig2 ((**B**), ×200), and GFAP ((**C**), ×200). The tumor tissue is located at the right side, and the peritumoral and intact tissues are at the left side of the photos; the border of the tumor is clear. Secondary antibody AlexaFluor 488 (green) + DAPI (blue nuclei).

To confirm the data of transcriptomic analysis, selective verification was performed by IHC and qPCR-RT methods. In IHC staining with antibodies to the oligodendrocyte marker protein Olig2 and the mature astrocyte marker GFAP, as well as the stem cell marker Sox2, a large number of Olig2^+^ and Sox2^+^ cells were observed in the tumor node relative to the peritumoral zone. At the same time, GFAP^+^ cells were not detected in the tumor (Figure 4).

**Figure 5:**
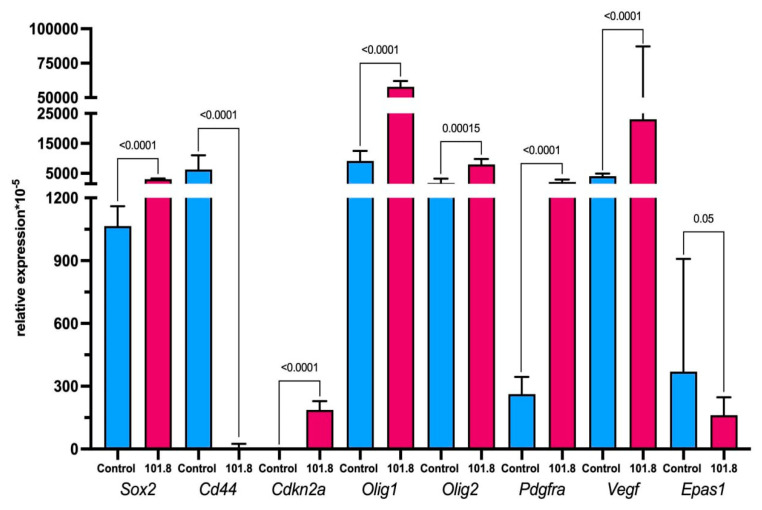
Relative expression levels of *Sox2*, *Cd44*, *Cdkn2a*, *Olig1*, *Olig2*, *Pdgfra*, *Vegf*, and *Epas1* in rat glioma 101.8 samples compared to intact brain tissue of the same region, with *n* = 5 for each group. *p* < 0.05. Mann–Whitney U-criteria. Data are shown as the median and interquartile range (Me; 25–75%).

According to qPCR-RT data, there was an increase in expression at the mRNA level for the stem cell marker *Sox2*, the oligodendrocyte markers *Olig1* and *Olig2*, cyclin-dependent kinase inhibitor 2A (*Cdkn2a*), platelet-derived growth factor receptor A (*Pdgfra*), and the angiogenesis-determining vascular growth factor *Vegf* in rat glioma 101.8 compared to the intact brain. In contrast, the expression levels of the hyaluronic acid receptor *Cd44* and the gene encoding hypoxia-inducible factor 2 alpha (HIF2a), *Epas1*, were lower in rat glioma 101.8 relative to healthy brain tissue (Figure 5). Thus, the data obtained by scRNA-seq were confirmed. According to them, rat glioma 101.8 tumor cells are predominantly of oligodendroglial origin with high expression of stemness and vascular growth factors.

### 2.5. Assessment of Tumor and Microenvironment Cells Identitied Using Sex-Linked Gene Expression Analysis

**Figure 6:**
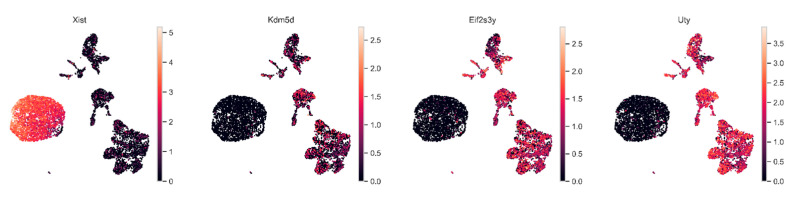
The expression of sex-linked genes in GB 101.8 samples obtained from male tumor recipient rats.

**Figure 7:**
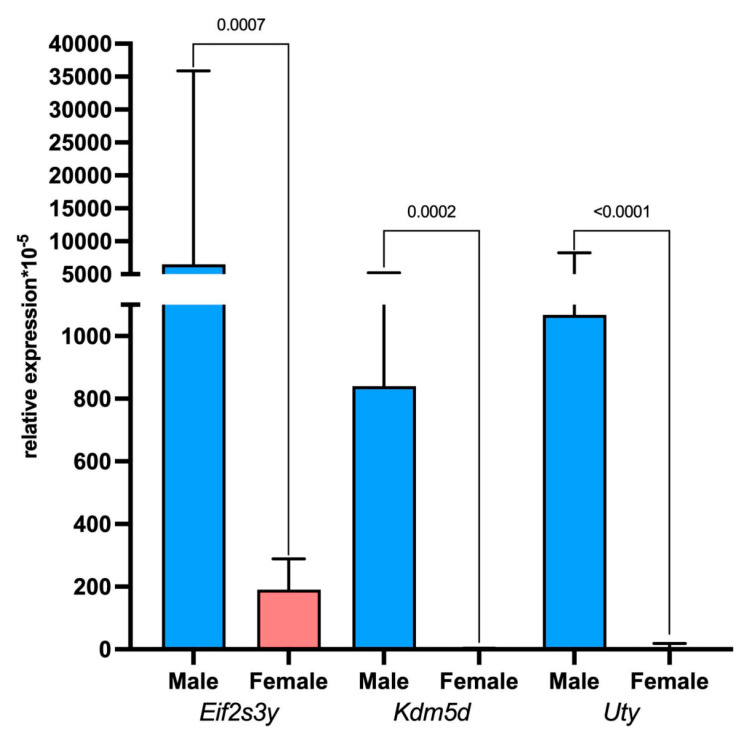
Relative expression levels of *Eif2s3y*, *Kdm5d*, and *Uty* (Y-chromosome genes) in the rat glioma 101.8 microenvironment in male and female tumor recipient rats. *p* < 0.05. Mann–Whitney U-test. Data are shown as the median and interquartile range (Me; 25–75%).

The transplantable rat glioma 101.8 tissue strain was initially induced in female Wistar rats; accordingly, the tumor cells exhibit an XX genotype and retain it almost completely (Figure 6. Some tumor cells lose sex chromosomes due to aneuploidy, which may confer a selective advantage or contribute to genomic instability, thereby promoting adaptation. Historically, the rat glioma 101.8 strain was sequentially transplanted mostly into male recipients. To discover the origin of the surrounding microenvironment, assessments of tumor cell identity and the microenvironment using sex-linked gene expression analysis were conducted based on the presence of X-linked (*Xist*) and Y-linked (*Kdm5d*, *Eif2s3y*, and *Uty*) genes. The data obtained via scRNA-seq showed that the XX genotype was detected in tumor cells of rat glioma 101.8, while the XY genotype was detected in cells of the tumor microenvironment (Figure 7).

**Figure 8:**
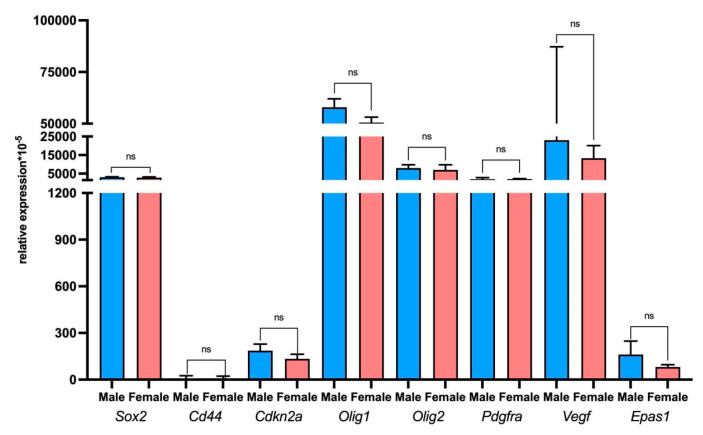
Relative expression levels of the *Sox2*, *Cd44*, *Cdkn2a*, *Olig1/2*, *PDGFRa*, *Vegf*, and *Epas1* genes in rat glioma 101.8 samples in male and female tumor recipient rats. ns—non-significant; *p* < 0.05. Mann–Whitney U-test. Data are shown as the median and interquartile range (Me; 25–75%).

According to the qPCR-RT study, no differences were found in the expression level of the tumor-associated genes *Sox2*, *Cd44*, *Cdkn2a*, *Olig1/2*, *PDGFRa*, *Vegf*, and *Epas1* in the rat glioma 101.8 tissue strain implanted in male and female rats (Figure 8).

**Figure 9:**
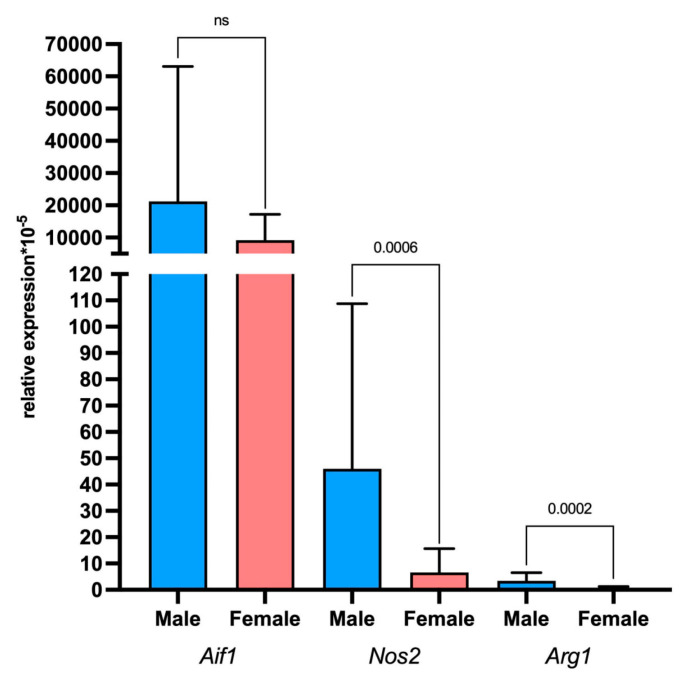
Relative expression levels of the *Aif1*, *Nos2*, and *Arg1* genes in rat glioma 101.8 samples in male and female tumor recipient rats. ns—non-significant; *p* < 0.05. Mann–Whitney U-test. Data are shown as the median and interquartile range (Me; 25–75%).

It was also found that the expression of the microglia marker gene *Aif1* in males and females did not differ. However, the expression of the M1 macrophage activation marker *Nos2* and the M2 macrophage activation marker *Arg1* was significantly upregulated in males in comparison to females (Figure 9).

## 3. Discussion

According to scRNA-seq and qPCR-RT data, rat glioma 101.8 tumor cells are characterized by high expression levels of the oligodendroglial markers *Olig1* and *Olig2*. The *Olig1/2* genes are members of the Olig family as well as major bHLH (basic helix-loop-helix) transcription factors regulating oligodendrocyte and neural cell differentiation [20]. In addition, high expression of *Olig1/2* was observed in oligodendrocyte progenitors [21]. *OLIG1/2* expression was observed mainly in oligodendroglioma of various degrees of malignancy including grades 3–4 [22,23]. At the same time, Olig1/2^+^ cells also occurred in glioblastoma [24], including low-differentiated cells, which led to aggressive tumor growth and a negative prognosis for patients [25].

Rat glioma 101.8 tumor cells were also characterized by high expression levels of *Pdgfra*. This gene encodes one of the most important markers of glioblastoma progression, second only to EGFR [26]. PDGFRa activates the PI3K/MAPK and PI3K/AKT signaling pathways and enhances proliferation, migration, and survival of tumor cells [26]. The gene is also associated with poor survival prognoses in patients with the H3K27M-mutant form of diffuse glioma [27]. However, its prognostic role in glioblastoma clinical outcomes is not yet established despite the frequency of its high expression. It is supposed that PDGFRa plays an important role in oligodendrocyte proliferation and differentiation [28]. Overall, *PDGFRa* is one of the targets for precision glioma therapy. Thus, the rat glioma 101.8 strain is quite relevant as a useful translational model for preclinical evaluation of targeted anti-tumor drugs aimed at reducing *PDGFRa* expression.

We found a high expression level of *Sox2* (sex-determining region Y-box 2) in rat glioma 101.8. It is a stem cell marker, a member of the SOX family, and one of the key genes in glioblastoma development. Its expression is associated with a highly aggressive tumor stem cell phenotype, and chemoresistance [29] was evaluated as a predictor of survival expectancy and therapy effectiveness [30,31]. Overexpression of *Sox2* together with *Nanog* and *Oct4* promotes the transformation of somatic cells into pluripotent cells. This marker is one of the key targets for therapy of various tumors including the CNS ones.

According to the literature, the activation of the pathways of stem cell growth (SOX2), oligodendrocyte transformation (OLIG1/2), and proliferation (PDGFRA) is the main characteristic of three types of human tumors: glioblastoma (wild-type IDH), H3K27M-mutant glioma (pediatric type), and IDH-mutant oligodendroglioma (1p/19q-codeletion). These three markers are also targets in glioblastoma precision therapy. Hence, rat glioma 101.8 is potentially a quite reliable preclinical model to evaluate the anti-tumor efficacy of SOX2-, OLIG1/2-, and PDGFRA-targeting therapy approaches [25,32].

As mentioned above, Olig2 is a marker not only of mature oligodendrocytes but also of oligo-like progenitor cells with a low differentiation degree. *OLIG2* is frequently highly expressed in grade 4 glioblastoma as well. It is worth noting that according to subcluster analysis of highly expressed genes, the following genes, in addition to *Mbp*, were also identified in the oligodendrocyte population: tubulin tyrosine ligase like 7 (*Ttll7*), whose high expression is observed in low-differentiated oligo-like progenitor cells [33]; amyloid precursor-like protein 1 (*Aplp1*), whose high expression was observed in highly malignant glioblastoma [34]; kallikrein 6 (*Klk6*), whose high expression is associated with a negative prognosis in glioblastoma due to chemo- and radioresistance [35]; and *Abca8*, a member of the ABC family involved in drug compound metabolism, whose high expression correlates with glioblastoma chemoresistance [36]. Data obtained via scRNA-seq analysis indicate the high proliferative and malignant potential of the rat glioma 101.8 tissue strain and the close correlation with signature signaling pathways found in malignant human glioma.

*Cdkn2a* is a tumor suppressor gene. Its silencing and decreased expression are associated with a poor prognosis and decreased sensitivity to cytostatics in highly malignant glioma [37]. Meanwhile, upregulation of *Cdkn2a* gene expression was reported for a number of glioma cell lines such as T98G, U87-MG, and SW1783 MG [38]. In this case, increased expression of this gene in rat glioma 101.8 presumably might be a consequence of the strain’s artificial origin and recurrent passaging.

Ubiquitin carboxyl-terminal hydrolase 33 (USP33) is an essential deubiquitinase that stabilizes the HIF-2a protein (encoded by the *Epas1* gene) via the ERK1/2-dependent mechanism to promote a hypoxia response in cancer cells. USP33 protein synthesis is primarily induced in glioma stem cells by hypoxia. It interacts with HIF-2a, leading to its stabilization through deubiquitination. The activation of ERK1/2 upon hypoxia promoted HIF-2a phosphorylation, enhancing its interaction with USP33 [39]. Apparently, there was a high amount of stabilized HIF-2a in rat glioma 101.8 tumor cells, which led to a decrease in *Epas1*’s mRNA expression level via a negative feedback mechanism. *Epas1* expression is correlated with the expression of another important marker of glioma stem cells (GSCs), the hyaluronic acid receptor gene *Cd44*. This gene is also associated with increased tumor cell migration and a poor life expectancy prognosis. An increase in its expression was noted in response to hypoxia [40]. However, high vascularization was observed in rat gliomas 101.8, which might lead to decreased expression of *Epas1* and *Cd44*. In the tumor, some cells also did not express *Ki67*, which is presumably related to the timing of the material obtained on the 18th day after tumor implantation. This is the terminal stage of 101.8 tumor growth, as the median life duration of animals after implantation of the rat glioma 101.8 tissue strain is 14 days [19].

The top 9 expressed genes in the tumor cell subcluster were identified as follows: cell adhesion molecule 2 (*Cadm2*), myelin basic protein (*Mbp*), Rho-GTPase 2 (*Rnd2*), synaptotagmin (*Syt4*), cyclin-dependent kinase inhibitor 1B (*Cdkn1b*), ankyrin repeat domain 37 (*Ankd37*), long non-coding RNA (*Cahm*), endoplasmic reticulum reticular oxygen reductase 1 alpha (*Ero1a*), and transmembrane glucose transporter protein (*Slc2a3*).

The predominant majority of rat glioma 101.8 tumor cells expressed *Cadm2*, *Cdkn1b*, and *Ero1a*. *Cadm2* is a regulatory gene encoding the CADM2 protein responsible for intercellular adhesion, and it is involved in tumor metastasis as part of the circHIAT1/miR-19a-3p/CADM2 signaling pathway [41]. Extremely high representation and expression of this gene was observed in the analyzed samples, indicating the aggressiveness of the rat glioma 101.8 strain. *Cdkn1b* is an inhibitor of cyclin-dependent kinase 1B. As a tumor suppressor, it is involved in the regulation of the cell cycle and proliferation in glioblastoma [42]. Deletion of the *CDKN1a/b* gene is typical for human glioma. However, the expression level of both *Cdkn1b* and *Cdkn1a* was quite high in rat glioma 101.8. Gliomas are characterized by a highly heterogeneous cellular composition, so the presence of cells with high expression of *Cdkn1a/b* and the absence of it in rat glioma 101.8 further confirm the diversity of gene signatures in this model and make it more relevant to human glioma than any other cell lines. *Ero1a* (endoplasmic reticulum reductase 1 alpha) is a negative prognostic marker in a variety of tumor types, including in EGFR-mutated forms of non-small cell lung cancer, colon cancer, and hepatocellular carcinoma [43].

Approximately half of the tumor cells or less demonstrated high expression of *Syt4*, *Ankd37*, and *Slc2a3*. Syt4 is a member of the synaptotagmin protein family, providing calcium-dependent vesicular neurotransmitter transport and exocytosis. According to the literature, Syt4 is a prognostic marker of low-grade malignant glioma [44]. There was low representation of cells expressing this gene in the studied samples. *Ankrd37* (ankyrin repeat domain 37) gene expression could be activated under hypoxia via the Hif-1a-dependent pathway, affecting autophagy, cell survival, and tumor progression [45]. *Slc2a3* (Glut3), a gene encoding a transmembrane glucose transporter protein, is actively expressed by neurons under physiological conditions. Its high expression is also observed in human glioblastoma tumor cells. Its increased expression has been shown to be associated with a negative prognosis of glioblastoma progression [46].

Minor cell populations expressed *Mbp*, *Rnd2*, and *Cahm* at high levels. *Mbp* (myelin basic/main protein) is an anti-oncogene responsible for suppressing tumor growth and exerting anti-proliferative effects [47]. The *Mbp* gene regulates myelin formation and is represented in rat C6 glioma cell lines and mature oligodendrocytes [48]. A low representation of cells expressing this gene possibly highlights the high proliferative potential of the rat glioma101.8 tissue strain. The *Rnd2* gene encodes a member of the Rho-GTPase family with no GTPase signaling activity. Under physiological conditions, the Rnd2 protein regulates the migration of neuronal precursors during an embryo’s brain development. Glioblastoma is characterized by high expression of *RND2*, which correlates with a negative prognosis [49]. *Cahm*, a gene encoding a corresponding long non-coding RNA, is localized near and related with the *Qki* gene, which is involved in inflammatory processes initiated through the activation of MAPK and neurokinin B pathways and might correlate with tumor malignization and a worse prognosis [50].

According to the transcriptome analysis of marker gene expression, the tumor microenvironment consisted of the following cell populations: dendritic cells, macrophages, B cells, T cells, and oligodendrocytes. Since the rat glioma 101.8 tissue strain was originally obtained from female rats, tumor cells carry X chromosomes with high expression of the sex-linked *XIST* gene [51]. In our work, the rat glioma 101.8 tissue strain with the XX genotype was orthotopically implanted into male rats. Accordingly, the tumor microenvironment carrying the XY genotype indicates the immunogenicity of this strain. These single-cell studies were confirmed by qPCR-RT, showing a high expression level of the Y-chromosome genes, namely *Eif2s3y*, *Kdm5d*, and *Uty*, in rat glioma 101.8 samples in male but not in female recipients.

The *EIF2S3Y* gene, located on the short arm of the Y chromosome, is highly expressed in the brain, regulates protein synthesis, and activates the Wnt/β-catenin pathway, promoting cell survival [52]. *Eif2s3y* expression is absent or occurs at a low level in female rats as well as in other female mammals. In our experiments which assess the origin of the tumor microenvironment during transplantation from an XY donor to either XX or XY recipients, *Eif2s3y* expression persisted in female recipients, albeit at a lower level than in male recipients. This could possibly be explained by cross-reactivity of the *Eif2s3x* gene since it is an X-linked homolog of *Eif2s3y* with approximately 80% homology. The observed expression might also be a result of epigenetic dysregulation, leading to activation due to amplification of the *Eif2s3x* homolog. Alternatively, a fragment of the Y chromosome containing *Eif2s3y* could integrate into autosomes or the X chromosome as a consequence of tumor genomic instability. In addition, XX tumor cells could absorb exosomes or microvesicles carrying *Eif2s3y* mRNA from male donor microenvironment cells during previous transplant passages.

The *KDM5D* gene, as well as the Y-linked one, encodes a protein demethylating H3K4. It could enhance genomic instability and promote tumor metastasis, and its high expression is linked to some diseases, including colorectal cancer [53]. The *UTY* gene, located only on the Y chromosome, regulates H3K27 demethylation and facilitates immune evasion in tumors via T cell dysfunction. These genes are considered potential targets for precision cancer therapy [54].

Our findings demonstrate that when tumor tissue (XX) along with its microenvironment (XY) is transplanted into male recipients, the microenvironment exclusively retains the XY genotype. Consequently, the recipient mounts an immune response against the transplanted host-derived tumor microenvironment due to MHC incompatibility. In addition, ischemia–reperfusion injury following tissue damage during transplantation may contribute to this process. As a result, the host-derived microenvironment is eliminated and replaced by the recipient’s own microenvironment. Both processes are able to initiate promotion of the pro-inflammatory microenvironment, which supports tumor growth during allograft engraftment. It is also consistent with the Virchow principle linking inflammation and tumorigenesis [55]. Subsequent development of the tumor depends on the interactions of the tumor tissues with the recipient.

Interestingly, the analysis of sex differences in oncogene expression did not reveal any differences between male and female rats. Regardless of the recipient’s sex, the tumor retains its molecular profile. These data indirectly indicate the preservation of the tumor strain’s authenticity during multiple transplantations and the weak influence of the microenvironment on the tumor genotype. A weak interaction between the implant and the recipient animal is a typical flaw of chemically induced tumor models; however, the stability of the strain allows us to rely on previously obtained data and plan further experiments on it.

There was also no difference in the expression level of the microglia marker *Aif1* between males and females. At the same time, *Nos2* and *Arg1*, markers of M1 and M2 macrophages, respectively, were upregulated in males compared to females. So, the tumor microenvironment is formed mainly by the tumor recipient organism. It was shown that the expression level of the M1 and M2 macrophage phenotypes and associated genes was higher in men suffering from glioblastoma compared to women [56]. Apparently, it is associated with sexual dimorphism of the immune response to the tumor [57], as well as the effect of estradiol on its receptors, which are highly expressed by microglia [58]. The study by Ochocka et al. showed that the representation of genes encoding MHCII in glioblastoma in men is higher than that in women according to scRNA-seq [59]. At the same time, the study by Tharp et al. showed that female TAM-MGs display stronger interferon signaling and cytotoxic T cell interactions, which should enhance anti-tumor immunity. As a result, immune therapy of glioblastoma showed higher efficacy in men, since the immune response in men is less pronounced [60]. Sex differences likely have a significant impact on the activation of the immune response, but the mechanisms are not fully understood. For a more detailed study, further studies are warranted.

Our analysis allowed us to identify the similarities and differences between the rat glioma 101.8 tissue strain and human glioblastoma. scRNA-seq and qPCR-RT revealed high expression of the *Olig1/2* genes. In human glioblastoma, these genes are activated in the proneural subtype of glioblastoma as well as the classical astrocytic type of glioblastoma [61]. The *Olig1/2* genes regulate cell differentiation and are responsible for chemotherapy resistance, so they are potential target genes for precision therapy [62]. Rat glioma 101.8 demonstrated high expression of the *Pdgfra* gene occurring in more than 10% of glioblastoma cases, mainly in proneural glioblastoma [63]. *Vegf* plays a key role in glioblastoma angiogenesis and was also highly expressed in rat glioma 101.8. It might be one of the key criteria for selecting this model for the development of anti-angiogenic therapy [64]. The *Sox2* gene, as a stemness marker, was highly expressed in the rat glioma 101.8 tissue strain as well, consistent with such findings in human glioblastoma [29]. In addition, our previous studies confirmed *Sox2* expression level as a marker of the effectiveness of rat glioma 101.8 therapy with the cytostatic doxorubicin [65]. It was previously shown that the rat glioma 101.8 tissue strain had increased expression of the multidrug resistance marker genes *Abcb1b* and *Mgmt*, which are targets for therapy, especially in recurrent glioblastoma [66,67].

Despite the difference in the molecular profile of human glioblastoma and the studied model, the morphology of rat glioma 101.8 and its phenotype have high similarity to diffuse high-malignancy glioma (grades 3–4). High-malignancy gliomas display high mitotic activity (astrocytoma grades 3–4) and extensive necrosis alongside high vascularization (glioblastoma grade 4). Histology of rat glioma 101.8 s demonstrated such a morphological picture. Although MRI and CT methods are only a tool for preliminary diagnosis, these types of instrumental research allow us to presume the tumor type and grade based on the contrast agent accumulation pattern. Rat glioma 101.8 acquired typical features of glioblastoma grade 4 in the form of the formation of a high-contrast ring along the periphery of the tumor with a non-contrast central zone. Some clinical cases show an uneven distribution of the contrast agent in the case of glioblastoma [68]. Rat glioma 101.8 is characterized by the formation of a “contrast ring” on day 7 of the experiment, which corresponds to human glioblastoma grade 4. However, the nature of the contrast distribution changes and is more reminiscent of astrocytoma grades 2–3 by the 18th day [68]. The accumulation of the contrast agent is directly related to tumor vascularization, which is an important sign in assessing tumor malignancy and predicting survival. Morphological examination of rat glioma 101.8 also revealed a high density of blood vessels of varying degrees of maturity. Thus, rat glioma 101.8 is similar to both grade 4 glioblastoma and grades 3–4 astrocytoma in its morphological and phenotypic features.

## 4. Materials and Methods

### 4.1. Animals

This study used 20 mature male and 5 female SPF Wistar rats (3–4 months old; weight: 220–250 g; source: Pushchino Laboratory Animal Nursery, Pushchino, Russia). Animals were housed under controlled conditions: a 12 h light/dark cycle with free access to food and water. All surgical procedures maintained aseptic standards. Rats exhibiting initial clinical signs of tumor growth, including decreased activity, paralysis, weight loss, weakness, and lethargy, were euthanized. All animal experiments complied with the Declaration of Helsinki and were approved by the Bioethics Commission (Protocol № 29(5); 8 November 2021) of the Avtsyn Research Institute of Human Morphology (FSBSI “Petrovsky National Research Center of Surgery”, Moscow, Russia). Rats were randomly divided into the following experimental groups with a minimum 5 animals in each group for the molecular experiment and 3 for the histological study.

The experiments were carried out in a short timeframe to avoid the influence of external factors and physiological changes in animals.

Different researchers were involved in the experiment. A.I.A. and K.V.V performed tumor implantation. A.M.K. randomly divided the animals into groups. A.V.S., E.A.M., and E.E.K. performed histological and molecular studies.

### 4.2. Revitalization and Transplantation of Tissue Derived from the GB 101.8 Tumor Strain

Before the main examination, the tumor tissue was revived. It was extracted from the cryopreservation unit (−196 °C); the ampoule with strain GB 101.8 was placed in a container with warm water at a temperature of 39 °C for rapid thawing. The sample was then centrifuged for 7 min at 250 g. After centrifugation, the supernatant was removed, and the precipitate was resuspended. Cells were carefully pipetted mechanically from the GB 101.8 tissue using 0.25% trypsin–EDTA (PanEco, Moscow, Russia) solution, and their viability and quantity were immediately determined by the LUNA-II Automated Cell Counter (Logos Biosystems, Seoul, Korea) with a 0.4% trypan blue solution. Cell viability was at least 96%. The skin of the parietal region of the rats’ heads was treated with antiseptic solutions. Each animal (*n* = 5) was injected with 20 µL of cell suspension (1 × 10^6^ cells) intracerebrally. A syringe with a G19 needle hole was used. To create a trepanation hole, a dental drill (Marathon, Malaga, Spain) with a diameter of 2 mm was used. The stereotactic coordinates are as follows: 2 mm right of the sagittal suture (Sutura sagittalis) and 2 mm caudal to the coronal suture (Sutura coronalis) at a depth of 3 mm from the dura mater in the hippocampal region (Fimbria hippocampi). The trepanation hole and skin wound were treated with an aseptic solution. The skin wound was sutured.

Freshly acquired tissue taken from mature tumors was transplanted into experimental animals (1 obtained tumor sample per 3 animals) as described above. All in vitro and in vivo procedures were conducted under aseptic conditions. Surgical interventions were performed under anesthesia using intraperitoneal injections of 10 mg/kg tiletamine hydrochloride (Zoletil, Virbac, Miribel, France) and 15 mg/kg xylazine hydrochloride (NITA-PHARM, Saratov, Russia).

### 4.3. MRI Imaging

On day 18 of tumor growth, MRI studies of the rat brain were performed on a BioSpec 70/30 USR MRI scanner (Bruker, Karlsruhe, Germany) with a constant magnetic field of 7.05 Tesla. Before scanning, the animals were injected through the tail vein with an MRI contrast agent containing gadolinium (Gd) (Gadovist^®^; Shering AG, Berlin, Germany) at a dose of 0.1 mmol/kg in 0.3 mL of 0.9% NaCl. For this purpose, the animals were previously immersed in anesthesia using Isoflurane 4.5% (SAMARTH life sciences, Thane, India) and oxygen 95%, after which they were transferred to a surgical table, and the animals’ heads were fixed in a mask where a mixture of isoflurane (1.5%) and oxygen (95%) was supplied. During the MRI study, the animals were also gas anesthetized with isoflurane (1.5%) and oxygen (95%). T_1_-weighted brain images were obtained in axial projection using a FLASH (Fast low-angle shot magnetic resonance imaging) gradient echo pulse sequence with the following basic scan parameters: TR = 200 ms, TE = 7.3 ms, spatial resolution: 0.13 × 0.13 mm, slice thickness: 0.8 mm, number of slices: 12, FA = 60°, number of accumulations: 4, and total scanning time: 2 min 41 s. Scanning was performed using two radiofrequency (RF) coils: a transmitting coil “birdcage” with an inner diameter of 72 mm and a receiving surface RF coil designed to study the brain of laboratory rats.

### 4.4. Preparation of Rat Glioma 101.8 Tissue Samples

To analyze rat glioma 101.8 tissue by single-cell RNA sequencing, animals (*n* = 20) were removed from the experiment on the 18th day of tumor growth and euthanized using an overdose of 25 mg/kg tilethamine hydrochloride (Zoletil, Virbac, Miribel, France). GB 101.8 tissue samples for single-cell seq analysis were obtained from two male Wistar rats (samples Gl1 and Gl2). The whole brain was extracted, and the tumor tissue was separated and crushed with surgical scalpels. Tumor cells were mechanically separated by pipetting and incubated for 5–10 min at a temperature of 37 °C with a 0.25% trypsin–EDTA (PanEco, Moscow, Russia) solution. Next, the tumor cells were separated on a cell sieve (Nunc, Hørning, Denmark) with a pore diameter of 40 µm (Corning, NY, USA). The cells were separated from the 0.25% trypsin–EDTA solution by centrifugation at 300× *g* (Eppendorf, Hamburg, Germany) for 5 min and resuspended in 1 mL of PBS (PanEco, Moscow, Russia) with 0.04% BSA (Invitrogen, Carlsbad, CA, USA). Further analysis of the resulting cell suspension was performed immediately.

DMEM high glucose was added to the suspension of the obtained tumor cells. The cells were counted using acridine orange and propidium iodide (Logos biosystems, Seoul, Korea) on a Luna II cell counter (Logos biosystems, Seoul, Korea). The number of cells was 3.4 × 10^7^ cells for the Gl1 sample and 2 × 10^7^ cells for the Gl2 sample. To increase the percentage of cell survival, a dead cell removal kit (Miltenyi biotec, Vianen, The Netherlands) was used. The cell survival rate in both samples was at least 30%.

The cells were centrifuged at 300 g; the supernatant fluid was removed, and Gl1 and Gl2 cell samples were resuspended in 300 and 200 µL of Dead Cell Removal microbeads (Miltenyi biotec, Vianen, Netherlands), respectively. The cell suspension was incubated for 15 min; then the volume of the mixtures was adjusted to 500 µL using a binding buffer (Zymo research, Irvine, CA, USA). Before using the MS column (Miltenyi biotec, Vianen, Netherlands), it was moistened with 500 µL of the binding buffer. The cell suspension was applied to the MS columns and washed with another 2 mL of the binding buffer. The cells were centrifuged at 300× *g*; the filler fluid was removed, and Gl1 and Gl2 cell samples were resuspended in PBS with 0.04% BSA. The obtained samples had concentrations of 2 × 10^6^ for G11 (viability—77%) and 2.3 × 10^6^ for Gl2 (viability—71%). Both samples were diluted to a final cell concentration of 1 × 10^6^/mL.

For RT-PCR studies, fragments of tumor tissue outside the necrosis zones were obtained from male (*n* = 5) and female (*n* = 5) rats, immediately placed in the IintactRNA fixator (Eurogen, Pushchino, Russia), and stored at a temperature of −70 °C until used.

For histological and immunohistochemical analyses, the whole brain was obtained from male rats (*n* = 3), fixed in 10% buffered formalin (Biovitrum, Moscow, Russia) for 72 h, and then placed in 70% ethyl alcohol. The samples were prepared using alcohols of increasing concentration in an automated device for histological examination of VIP5Jr tissue (Sakura, Tokyo, Japan), and then placed in the Histomix paraffin medium (Biovitrum, Moscow, Russia), and histological sections 5–7 µm thick were made on an HM340E microtome (Thermo-scientific, Giessen, Germany). For histological examination, the sections were stained with hematoxylin and eosin.

### 4.5. Single-Cell RNA Sequencing of Rat Glioma 101.8 Tumor Samples

For each experimental sample, 1.65 × 10^4^ cells (70% viability) in PBS with the 0.04% BSA solution were taken into the scRNA-seq library preparation experiment. The scRNA-seq libraries were made according to the Chromium Next GEM Single Cell 3’ (Dual Index) v3.1 User Protocol (10× Genomics, Pleasanton, CA, USA). The GEM particles were generated on Chromium iX/X (10× Genomics, Pleasanton, CA, USA). The amplification steps were performed on the RT-PCR machine QuantGene 9600 (Bioer, Hangzhou, China). The quality check of the scRNA-seq libraries was performed using the Fluo-100 fluorimeter (Allsheng, Hangzhou, China) and the QSep400 Bio-Fragment Analyzer (BiOptic Inc., New Taipei City, Taiwan). The scRNA-seq libraries were sequenced on the GenolabM platform (Genemind, Shenzhen, China) with the following program: 28 cycles for read 1 and 90 cycles for read 2.

### 4.6. PCR-RT

mRNA was isolated from rat glioma 101.8 tissue samples of male (*n* = 5) and female (*n* = 5) rats after tumor cell injection and control male rats (*n* = 5) using the RNeasy kit (Qiagen, Hilden, Germany). An aliquot of homogenized brain hemisphere tissue was used as a source of intact brain tissue. cDNA synthesis was performed using the SK-021 kit (Eurogen, Pushchino, Russia). The mRNA expression level was assessed on a DTprime 4M1 device (DNA Technology, Moscow, Russia) using a PK-147L kit with the intercalating dye SYBR Green (Eurogen, Pushchino, Russia). All the primers’ sequences were selected precisely for rat species using the online software Primer-BLAST (Table S4). All samples were used for further analysis.

### 4.7. Histological Examination

Histological examination and preparation of micrographs of hematoxylin and eosin (H&E)-stained brain sections from rats bearing rat glioma 101.8 implanted tissue were performed using a Zeiss Axioplan 2 light microscope and a AxioCam HRc camera (Carl Zeiss, Oberkochen, Germany).

### 4.8. Immunohistochemical Analysis

After dewaxing, histological sections of the brain with a tumor (*n* = 3) were restored according to the standard protocol by epitope boiling in a citrate buffer. The solution was cooled and washed twice in PBS for 5 min (pH 7.2–7.4). A blocking protein buffer solution with 1% BSA (Invitrogen, Carlsbad, CA, USA) was applied, and the following primary antibodies (100 µL, 1:100) were used: anti-Olig2 (GB11766, Servicebio, Wuhan, China), anti-Sox2 (GB11249, Servicebio, Wuhan, China), and anti-GFAP (GB12096, Servicebio, Wuhan, China). The slices were incubated with primary antibodies at a temperature of 4 °C overnight. The next day, they were washed twice in PBS for 5 min, and the following secondary antibodies were applied: Donkey anti-Rabbit AlexaFluor 488 (100 µL, 1:500, 711-165-152, Jackson ImmunoResearch, West Grove, PA, USA) and Donkey anti-Mouse AlexaFluor 488 (100 µL, 1:500, 718-545-150, Jackson ImmunoResearch, West Grove, PA, USA). Samples were incubated for 2 h at room temperature in the dark. They were then washed twice in PBS for 5 min, and a solution of the nuclear dye DAPI (100 µL, 0.1 µg/mL) (Invitrogen, Carlsbad, CA, USA) was applied, followed by incubation in the dark for 10 min. The finished histological sections were enclosed in an Anti-Fade Fluorescence Mounting Medium (Abcam, Cambridge, UK) and stored at a temperature of 4 °C. Fluorescence microscopy of the preparations (Zeiss Axioplan 2 and AxioCam HRc cameras (Zeiss, Oberkochen, Germany)) in the green and blue spectra was performed.

### 4.9. Statistical Analysis

The data obtained in our study were analyzed using GraphPad Prism v.8.4.3 (GraphPad Software LLC, San Diego, CA, USA), StatTech v.2.8.8 (StatTech, Moscow, Russia), and Statistica 8.0 (StatSoft Inc., Aliso Viejo, CA, USA). The normality of the quantitative data distribution was assessed using the Shapiro–Wilk test, the Kolmogorov–Smirnov test, and visual evaluation of Q-Q plots. Hazard ratios (HRs) with 95% confidence intervals were calculated using Cox proportional hazard regression. For group comparisons the Mann–Whitney U-test was applied since there were non-normally distributed data. Multiple comparisons were analyzed using the Kruskal–Wallis test followed by Dunn’s post hoc test for pairwise comparisons. Data are shown as the median and interquartile range (Me; 25–75%). Differences were considered statistically significant at *p* < 0.05.

We aligned raw sequencing data in FASTQ format to the gray rat (Rattus norvegicus) reference genome mRatBN7.2 using Cell Ranger 7.0.1 (10× Genomics) to generate gene expression matrices. scRNA-seq data analysis was performed using Scanpy (v1.9.5). Preprocessing included quality control filtering of cells and genes, excluding mitochondrial and ribosomal genes, and doublet removal. Doublets were detected using Scrublet with automatic threshold determination and subsequently removed based on Scrublet scores and elevated expression of multiple cell-type markers. Cell filtering thresholds were set as follows: 1500 ≤ *n*_genes_by_counts ≤ 8000; 1000 ≤ total_counts ≤ 60,000; and pct_counts_mt < 5%. Only genes detected in at least 3 cells were retained. Data were normalized to 10,000 reads per cell using Scanpy’s normalize_total method with subsequent log1p transformation. Principal component analysis (PCA) was performed on the top 3000 highly variable genes. Cell clustering was conducted using the Leiden algorithm (resolution = 1.4), with UMAP for dimensionality reduction visualization. Batch effect correction was performed using Harmony. Tumor cells were identified through aneuploidy detection with SCEVAN. Cluster-specific differentially expressed genes (DEGs) were identified using Scanpy’s rank_genes_groups function (Wilcoxon rank-sum test) with significance thresholds set at |log2 fold change| > 1 and adjusted *p*-values < 0.05, applying Bonferroni correction. Final cluster annotation was performed manually based on DEG expression patterns and established marker genes from the literature.

## 5. Conclusions

Using the scRNA-seq method, the experimental rat glioma 101.8 tissue strain was characterized. The cellular composition of the strain was determined, as well as the major gene signatures of each tumor cell population. Dendritic cells, macrophages, and a large number of B and T lymphocytes, in particular CD8^+^ T-cytotoxic cells, were detected in the tumor microenvironment, which allows us to investigate new approaches to CAR-T therapy of glioblastomas in this model. The top 9 most expressed gene signatures were identified in the tumor cell population, including those encoding proteins of cell adhesion, vesicular transport, kinase inhibitors, glucose transporter proteins, adaptive responses to hypoxia, etc. The data obtained proves the usefulness of this model for the development of methods of targeted therapy and personalized medicine.

### Limitations of the Study

The study has a number of limitations. Due to the high financial capacity, only two samples of the rat glioma 101.8 tissue strain were analyzed. The data obtained by comparison were identical, allowing us to suggest high reproducibility of the model. In addition, tumor samples were obtained on the 18th day after implantation, i.e., late in the course of disease development, as described earlier. At this timepoint, extensive necrosis is morphologically defined, which presumably affected cell proliferation, as confirmed by the low expression level of *Mki67*. In further studies, it would be advisable to observe the process dynamics and examine samples at earlier times of tumor growth after implantation.

## Figures and Tables

**Table 1:** Marker genes for cell type annotation.

Cell Type	Marker Genes
Glioma cells	*Sox2*, *Olig2*, *Pdgfra*, *Cspg4*, and *Nes*
B lymphocytes	*Cd79a*, *Ms4a1*, *Pax5*, *Cd24*, *Cd27*, and *Cd19*
Plasmacytoid dendritic cells	*Bst2*, *Tcf4*, *Irf7*, and *Ly6c*
Macrophages	*Cd68*, *Adgre1*, *Mrc1*, *Ccl2*, *Cd86*, and *Itgam*
Dendritic cells	*Clec9A*, *Cadm1*, *Xcr1*, *Itgax*, *Ccr7*, *Cd80*, and *Cd86*
Plasma cells	*Mzb1* and *Prdm1*
Microglia	*Itgam*, *Ptprc*, *Cx3cr1*, *P2ry12*, and *Hexb*
T lymphocytes	*Cd3e*, *Cd4*, *Cd8a*, *Foxp3*, *Il2*, *Icos*, *Gzmb*, and *Prf1*
T-regulatory lymphocytes	*Foxp3*, *Il2rA*, *Tgfb1*, *Ctla4*, *Ikzf2*, *Tigit*, *Lag3*, and *Pdcd1*
Proliferating cells	*Mki67*, *Top2a*, and *Pcna*

## Data Availability

All data analyzed during this study are included in this article and available from the corresponding author upon reasonable request.

## Supplementary Materials

The following supporting information can be downloaded at: https://www.mdpi.com/article/10.3390/ijms26188992/s1.
