# Supplementary material for: Rat Glioma 101.8 Tissue Strain: Molecular and Morphological Features
Source: Int J Mol Sci. 2025 Sep 15;26(18):8992. doi: 10.3390/ijms26188992 (PMC12469387; doi:10.3390/ijms26188992)
Supplement: Supplementary file 1 [file ijms-26-08992-s001.zip › ijms-3833228-supplementary/ijms-3833228-supplementary proofed/Table S1.pdf]

Table S1. Clusters distribution of cell populations depending on marker gene representation (integrated analysis of two samples).

Cell subpopulation	Absolute number of cells	Relative number of cells, %
Tumor cells	2612	35.3
Macrophages	767	10.4
Proliferating tumor cells	670	9.1
B-lymphocytes	599	8.1
$CD4^+$ T-lymphocytes	524	7.1
$CD8^+$ T-lymphocytes	443	6
$CD4^+$ Proliferating T-lymphocytes	404	5.5
$CD4^+$ T-regulatory lymphocytes	340	4.6
$CD4^+$ Proliferating T-regulatory lymphocytes	300	4.1
$CD8^+$ Cytotoxic T- lymphocytes	209	2.8
$CD8^+$ Proliferating cytotoxic T lymphocytes	158	2.1
Plasma cells	129	1.7

Dendritic cells	87	1.2
Plasmacytoid dendritic cells	82	1.1
$CD86^+$ activated dendritic cells	55	0.7
Oligodendrocytes	16	0.2
