# Supplementary material for: Rat Glioma 101.8 Tissue Strain: Molecular and Morphological Features
Source: Int J Mol Sci. 2025 Sep 15;26(18):8992. doi: 10.3390/ijms26188992 (PMC12469387; doi:10.3390/ijms26188992)
Supplement: Supplementary file 1 [file ijms-26-08992-s001.zip › ijms-3833228-supplementary/ijms-3833228-supplementary proofed/Table S2.pdf]

Table S2. Top-5 differentially expressed genes, signaling pathways, and biological processes for each glioma cell cluster

Cluster	Genes	Expression change (relative to the average expression among the other clusters)	
		Number of times	Logfold change
B-lymphocytes	*EBF1*	335.6	8.4
	CD79A	102.9	6.7
	PKIB	49.7	5.6
	LOC120093161	48.6	5.6
	BANK1	263.6	8.0
CD4+ T-lymphocytes	*CD28*	17.5	4.1
	LOC102554731	19.5	4.3
	RORA	15.1	3.9
	FYB1	9.9	3.3
	CD247	13.0	3.7

CD4+ T-regulatory lymphocytes	*ICOS*	23.7	4.6
	IFI27L2B	22.4	4.5
	IL2RA	32.6	5.0
	MAF	19.5	4.3
	LOC100910973	13.5	3.8
CD8+ T-lymphocytes	*ZFP36L2*	7.5	2.9
	SATB1	18.7	4.2
	PRKCQ	15.9	4.0
	GZMM	59.6	5.9
	SKAP1	12.0	3.6
CD86+ activated dendritic cells	*TMEM123*	29.6	4.9
	CAB39	13.2	3.7
	LSP1	32.5	5.0
	TBC1D4	29.1	4.9

	CRIP1	29.7	4.9
CD8+ T-cytotoxic lymphocytes	*NKG7*	45.3	5.5
	CD8A	22.5	4.5
	RUNX2	12.8	3.7
	PRKCH	10.4	3.4
	CCL4	37.3	5.2
Dendritic cells	*S100A11*	12.8	3.7
	LSP1	13.4	3.7
	CST3	27.0	4.8
	IFI30	18.1	4.2
	TMSB10	5.5	2.4
Macrophages	*DOCK4*	31.0	5.0
	RT1-DB1	33.1	5.0
	LYZ2	96.5	6.6

	FCER1G	161.4	7.3
	BCL2A1	16.0	4.0
Oligodendrocytes	*APLP1*	155.8	7.3
	MBP	91.6	6.5
	ABCA8A	805.2	9.7
	TTLL7	39.0	5.3
	KLK6	15339.9	13.9
Proliferating dendritic cells	*SIGLECH*	5321.5	12.4
	TCF4	24.3	4.6
	IFI30	52.0	5.7
	TYROBP	104.4	6.7
	FLT3	157.7	7.3
Plasma cells	*JCHAIN*	879.6	9.8
	LOC120093161	254.7	8.0

	MZB1	655.4	9.4
	HSP90B1	17.2	4.1
	SDF2L1	19.1	4.3
Proliferating CD4+ T-lymphocytes	*PFN1*	3.4	1.7
	S100A4	17.2	4.1
	CRIP1	9.3	3.2
	CORO1A	7.4	2.9
	LOC102554731	15.3	3.9
Proliferating CD4+ T-regulatory lymphocytes	*PFN1*	3.9	2.0
	IL2RA	20.3	4.3
	ICOS	13.7	3.8
	TMSB10	2.8	1.5
	HMGB2	7.2	2.9
	HMGB2	9.2	3.2

Proliferating cytotoxic CD8+ T-lymphocytes	*NKG7*	28.3	4.8
	MKI67	12.7	3.7
	HMGB2L1	11.7	3.5
	CD8A	15.0	3.9
Proliferating tumor cells	*PRC1*	10.6	3.4
	TOP2A	13.8	3.8
	RACGAP1	9.3	3.2
	CENPF	11.3	3.5
	TPX2	10.3	3.4
Tumor cells	*SEMA3E*	7.5	2.9
	LOC100912167	15.6	4.0
	JAKMIP2	6.8	2.8
	TIMP3	8.6	3.1
	MAP1B	19.3	4.3
