# Supplementary material for: Rat Glioma 101.8 Tissue Strain: Molecular and Morphological Features
Source: Int J Mol Sci. 2025 Sep 15;26(18):8992. doi: 10.3390/ijms26188992 (PMC12469387; doi:10.3390/ijms26188992)
Supplement: Supplementary file 1 [file ijms-26-08992-s001.zip › ijms-3833228-supplementary/ijms-3833228-supplementary proofed/Table S3.pdf]

Table S3. Top-10 most highly expressed genes in different tumor cluster subtypes

Tumor cluster cell subtypes	Gene	Logfold change. arb. units	Expression upregulation. arb. units
1. Tumor cells	*CD63*	1.2	2.2
	CCND1	1.9	3.7
	TMEM100	1.7	3.3
	S100A10	1.2	2.3
	EMP3	1.2	2.3
	VGF	2.2	4.5
	IGFBP3	1.7	3.4
	NKAIN1	1.2	2.4
	PHLDA1	1.2	2.2
	EPCAM	1.1	2.2
2. Proliferating tumor cells I	*TOP2A*	5.0	32.1

	MKI67	4.2	18.6
	PRC1	3.6	12.4
	SMC4	3.0	8.1
	CENPF	4.2	18.6
	TPX2	3.9	15.4
	UBE2C	4.2	17.9
	RACGAP1	3.4	10.3
	NUSAP1	4.4	21.6
	SGO2	4.0	16.4
3. Proliferating tumor cells II	*MCM6*	3.2	9.1
	PCNA	2.4	5.4
	ATAD2	2.7	6.5
	UNG	3.7	13.1

	PCLAF	3.6	12.0
	POLA1	2.3	4.9
	GMNN	2.5	5.6
	DUT	2.2	4.6
	UHRF1	2.7	6.5
	RANBP1	2.1	4.2
4. Mbp+ oligodendron-like cells	*MBP*	4.9	29.9
	GPR17	4.5	23.3
	CNP	3.2	9.5
	TNR	3.0	8.2
	TUBB4A	5.0	31.5
	NFASC	3.7	13.1
	TNS3	3.1	8.3

5. Syt4+ tumor cells	*HSPB1*	2.2	4.5
	SYT4	1.7	3.2
	CTTNBP2NL	1.3	2.5
	HSPA5	1.2	2.3
	S100A10	1.1	2.2
	GADD45G	2.0	4.1
	PPP1R10	1.3	2.4
	STYXL1	1.6	3.0
	SDF2L1	1.6	2.9
	DNAJB11	1.1	2.1
6. Proliferating Syt4+ tumor cells	*PTTG1*	3.5	11.5
	JPT1	1.5	2.8
	BIRC5	2.7	6.5

	PTMS	1.6	3.1
	TUBA1A	1.6	2.9
	H2AZ1	1.7	3.2
	HDGF	1.3	2.5
	HMGB1	1.5	2.8
	CCNB2	3.0	7.9
	NUCKS1	1.2	2.3
7. Cahm+ tumor cells	*CAHM*	2.1	4.4
	DYNLT2	2.2	4.5
	LOC108352411	1.8	3.4
	SLC2A1	1.6	3.0
	HSPB1	1.5	2.8
	PPP1R10	1.6	3.0

	BTD	1.5	2.8
	INTS12	1.4	2.6
	LOC102546620	2.7	6.4
	LOC100912167	1.4	2.6
8. Cadm+ tumor cells	*LHFPL3*	1.2	2.3
	MDGA2	1.4	2.7
	CADM2	1.6	3.1
	NLGN1	1.4	2.6
	XYLT1	1.4	2.7
	TNR	1.4	2.6
	ZEB1	1.0	2.0
	CHST11	1.2	2.4
	LOC102546835	1.7	3.2

	NXPH1	1.3	2.4
9. Ankrd37+ tumor cells	*ANKRD37*	2.8	7.1
	CDKN1B	2.1	4.4
	EIF1	1.0	2.0
	VEGFA	2.3	4.8
	ERO1A	2.2	4.5
	DNAJC5	1.6	3.0
	SLC2A3	2.3	5.0
	GAPDH	1.2	2.3
	MIF	1.0	2.0
	LOC108352161	1.4	2.7

*relative to the average expression of the other clusters
