# Supplementary material for: Rat Glioma 101.8 Tissue Strain: Molecular and Morphological Features
Source: Int J Mol Sci. 2025 Sep 15;26(18):8992. doi: 10.3390/ijms26188992 (PMC12469387; doi:10.3390/ijms26188992)
Supplement: Supplementary file 1 [file ijms-26-08992-s001.zip › ijms-3833228-supplementary/ijms-3833228-supplementary proofed/Table S4.pdf]

Table S4. Sequence of primers used to determine the expression level of the corresponding genes.

Gene	Forward primer	Reverse primer
Kdm5d	GGGGTGTATGGGGAGGTGTGCCCAA A	CTGCTCACCACCAGCCTCCCCAGAA
Eif2s3y	GGGGGTGTGTAGTAGCTGGTGGTGGT AGTA	ATCTGCTCGGCGGCATAATGTTGGGGGT
Uty	GTGGGGCCTTGCTTGCTGGAGAGCTC TCTCTT	GGAGCAGGCAGGCCTGGTACTGTGTG
Sox2	TCCATGGGGGCTCTCTGTGGTGGTCA A	CATGTGCAGTCTCTACTGGGCG
Cd44	TTTATTATTGGGGGAGCACCACCCTG GC	AGGGGTAGTAGTAGTCATCAAGGCTGTGT
Cdkn2a	GTACCCCCCGATACAGGTGATGATGA T	GATACCGCAAATACCGCACG
Olig1	CTGTCTTTCAGGCTCGCACA	CGCTACGACATGCCTTGCTA
Olig2	TGAAGATCAACAGCCGCGAG	CCCCGTAAATCTCGCTCACC
Pdgfra	TGAGCGCGCAGATAGTGGCTA	GCCAATATATCATCCATCATCATGTC
Vegf	GCAGACACAGTGCTCCAGC	CCTGGGGGACCACCACTTGGCAT
Epas1	ACCTGATTGATTGTGTGCGGGACTC	GCTCCGAGAGCTGCTGCTCCTTT
Aif1	AGCAAGGATTTGCAGGGAGG	TGAAGGCCTCCAGTTTGGAC
Nos2	CGCTGGTTTGAAACTTCTCAG	GGCAAGCCATGTCTGTGAC

Arg1	GGATGAGCATGAGCTCCAAG	GCCAGCTGTTCATTGGCTT
Gapdh	CAGGGGGCTGCTGCCTTCTTCTCTTG TGTGTG	GCCTTGCTGACTGTGTGCCGTTGTTGAA
